# Ongoing Excess Hospitalizations for Severe Pediatric Group A Streptococcal Disease in 2023–2024—A Single-Center Report

**DOI:** 10.3390/idr16050067

**Published:** 2024-09-02

**Authors:** Nina Schöbi, Andrea Duppenthaler, Matthias Horn, Andreas Bartenstein, Kristina Keitel, Matthias V. Kopp, Philipp K. A. Agyeman, Christoph Aebi

**Affiliations:** 1 Division of Pediatric Infectious Disease, Department of Pediatrics, Bern University Hospital, Inselspital, University of Bern, CH-3010 Bern, Switzerland; nina.schoebi@insel.ch (N.S.); andrea.duppenthaler@insel.ch (A.D.); matthias.horn@insel.ch (M.H.); matthias.kopp@insel.ch (M.V.K.); philipp.agyeman@insel.ch (P.K.A.A.); 2 Department of Pediatric Surgery, Bern University Hospital, Inselspital, University of Bern, CH-3010 Bern, Switzerland; andreas.bartenstein@insel.ch; 3 Pediatric Emergency Center, Department of Pediatrics, Bern University Hospital, Inselspital, University of Bern, CH-3010 Bern, Switzerland; kristina.keitel@insel.ch; 4 Airway Research Center North (ARCN), University of Lübeck, D-23562 Lübeck, Germany

**Keywords:** *Streptococcus pyogenes*, invasive group A streptococcus, iGAS, child, outbreak, influenza

## Abstract

A Europe-wide outbreak of invasive pediatric group A streptococcal infections (iGAS) began in fall 2022. Here, we report the evolution of GAS hospitalizations in children and adolescents during the second outbreak year in 2023–2024 at a tertiary center in Switzerland. Using prospective monitoring of all in-patient GAS cases below 16 years of age, including those with iGAS, we compared case frequencies and clinical characteristics in three time periods (2013–2020; 2022–2023; 2023–2024). Annual GAS hospitalizations increased from a median of 25 cases (range 11–28) in 2013–2020 to 89 and 63 cases, respectively, in 2022–2023 and 2023–2024. iGAS cases evolved similarly (2013–2020, 4 cases (3–8); 2022–2023, 32 cases; 2023–2024, 21 cases). The decline in cases from 2022–2023 to 2023–2024 included all types of GAS organ involvement, except suppurative infections in the head area, which remained largely unchanged (48 vs. 45 cases). Pleural empyema declined from 13 to 7 cases, possibly explained by a poor overlap of the GAS and influenza curves, respectively, in 2023–2024 compared to 2022–2023. These data document the prolongation of the GAS outbreak into its second winter season in 2023–2024.

## 1. Introduction

Winter 2022–2023 witnessed an increase in severe group A streptococcal (*Streptococcus pyogenes*, GAS) infections in children and adolescents in many parts of Europe [1] and abroad [2,3]. Their cause is incompletely understood, but the disrupted circulation of respiratory tract pathogens brought about by the COVID-19 pandemic and the appearance of genetically distinct GAS clones were likely contributors. Most notable was the emergence of previously unknown clades (e.g., M1_UK_, M1_DK_) [4,5]. Accordingly, it was difficult to predict how case frequencies would evolve from mid-2023 onwards and the scientific community was left with closely monitoring GAS disease activity in order to gain a better understanding of this outbreak.

We recently reported epidemiologic and clinical data on pediatric GAS disease requiring hospital admission to our tertiary care center in Switzerland over a 10-year observation period ending on 30 June 2023 [6]. Our data reflected developments similarly reported from numerous other sites across Europe. Apart from a threefold increase in overall GAS hospitalizations in 2022–2023 compared with the prepandemic years 2013–2020, we noted a six-fold increase in invasive GAS infections (iGAS). Among these, pleural empyema stood out as the most dramatic surge, and an analysis of concomitant viral circulation identified influenza A and B as the viral species circulating synchronously in our in-patient population. A concomitant increase in GAS pleural empyema cases had been reported in many other sites in Europe [7].

In this report, we describe the evolution of GAS hospitalizations in children and adolescents during the most recent 2023–2024 epidemiologic year, ending on 30 June 2024.

## 2. Methods

Our institution is the only provider of pediatric in-patient services for a population of 1.2 million inhabitants (14% of the Swiss population). We monitored all hospitalizations to our institution with GAS infection as the main discharge diagnosis in patients below 16 years of age, as previously described [6]. An epidemiologic year was defined as a period of one year beginning on 1 July. Comparator cohorts of the 2023–2024 cohort consisted of cases in the prepandemic period extending from 1 July 2013 to 30 June 2020 and the 2022–2023 cohort, respectively. iGAS was defined as previously described (Table S1, Supplementary Data File [6]). Briefly, iGAS required (1) the identification of GAS in a normally sterile body site (by culture, PCR, antigen test) such as blood, cerebrospinal fluid or aspirate from a body cavity (e.g., pleural space, joint) or surgically sampled deep tissue or (2) the presence of toxic shock syndrome [8] or necrotizing fasciitis. For GAS organ involvement, the following definitions were used: The term “head” infection comprised suppurative GAS infections of the eye (e.g., orbital cellulitis), ear (e.g., mastoiditis), throat (e.g., peritonsillar or retropharyngeal abscess) or neck (e.g., cervical lymphadenitis). “Skin and soft tissue” infection included abscesses, cellulitis or necrotizing fasciitis. “Lower respiratory tract” infection denoted pleural empyema or bacteremic pneumonia. “Bone, joint, muscle” infections consisted of osteomyelitis, septic arthritis or bacterial myositis. “Systemic-onset” infection denoted invasive GAS infections without an identified focal site of infection other than acute pharyngitis. The clinical parameters extracted for each case are listed in Table 1. In-house viral surveillance of nasopharyngeal secretions of all patients admitted for acute respiratory tract illness was carried out using a direct immunofluorescence panel (Influenza A and B, rhino/enterovirus, Respiratory Syncytial Virus [RSV], parainfluenza types 1–3, human metapneumovirus, adenovirus) [9] and SARS-CoV-2 PCR (from 2020 onwards) until 30 November 2023. This was changed to a four-plex PCR panel (Influenza A, Influenza B, RSV, SARS-CoV-2) on 1 December 2023. For statistical analyses of ordinal data, we used the Mann–Whitney U test or the Kruskal–Wallis test as appropriate. Contingency tables (2 × 2 and 2 × 3) were used for frequency data. VassarStats (www.vassarstats.net, accessed on 10 July 2024) and GraphPad Prism version 10.0.0 for Windows (GraphPad Software, Boston, MA, USA, www.graphpad.com) were used for analyses and figure drawing.

## 3. Results

**Figure 1:**
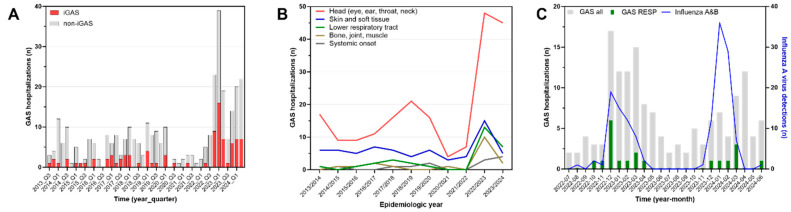
(**A**) depicts the quarterly case frequencies of GAS hospitalizations without (gray bars) and with (red bars) invasive group A streptococcal disease (iGAS). (**B**) shows the annual GAS case frequencies according to the primary organ system involved. (**C**) illustrates the monthly occurrence in 2022–2024 of all GAS cases (gray bars), GAS lower respiratory tract infections (green bars) and influenza virus detection frequencies among all patients admitted for an acute respiratory illness to our institution (blue line).

The evolution of case counts over time since 1 July 2013 is depicted in Figure 1A. In the 2023–2024 epidemiologic year, these were lower (63 cases) than in 2022–2023 (89 cases) but continued to exceed the prepandemic annual median (25 cases, range 17–28) by 2.5-fold (Table 1). iGAS cases evolved similarly (2013–2020, median 4 cases, range 3–8; 2022–2023, 32 cases; 2023–2024, 21 cases). A total of 19 cases, including 3 iGAS cases, occurred intrapandemically between 1 July 2020 and 30 June 2022. The lowest quarterly activity during the entire outbreak period from 2022 to 2024 was observed in the third quarter of 2023 (seven cases). In 2013–2020, the third quarter median was three cases (range 2–10). In terms of clinical manifestations, resolving the time series since 2013 according to the primary organ system involved resulted in the findings shown in Table 1 and Figure 1B. GAS infections of the head accounted for 61% of cases overall and fluctuated considerably. After the major surge in 2022–2023, with 48 cases, the case count was 45 in 2023–2024. In contrast, skin and soft tissue infections, infections of bone, joints and muscles and lower respiratory tract infections promptly declined to lower figures after the peak in 2022–2023. In both outbreak years combined, lower respiratory tract disease predominantly consisted of pleural empyema (17 cases), while bacteremic pneumonia without empyema was infrequent (3 cases). Skin and soft tissue infections observed during the entire study period mostly consisted of cellulitis or skin abscesses. Necrotizing fasciitis was rare (two cases in 2022–2023, both associated with varicella).

While the case frequencies in the two outbreak years strongly exceeded the case counts in the preceding decade, there was only limited evidence for greater case severity among hospitalized patients. A significantly greater proportion of cases was bacteremic and fulfilled the criteria of iGAS (Table 1). This, however, did not translate to a greater proportion of cases being diagnosed with septic and/or toxic shock compared with the prepandemic period. Accordingly, there were no significant differences in key outcome markers listed in Table 1, which remained stable throughout the entire observation period. An exception was the non-significant increase in inotrope use among ICU patients in 2023–2024.

Temporal association with the circulation of influenza viruses is examined in Figure 1C. While evolving synchronously with GAS counts in 2022–2023, the epidemic curves in 2023–2024 were discordant. In this year, the respective monthly peak frequencies occurred three months apart. Green bars identifying lower respiratory tract infections occurred almost exclusively during the influenza epidemics. The seasonal activities of RSV and SARS-CoV-2 did not correlate with GAS cases (Figure S1, Supplementary Data File).

## 4. Discussion and Conclusions

The data presented here provide evidence of an ongoing excessive occurrence of severe pediatric GAS infections including iGAS in the second year following the abrupt onset of the outbreak in fall of 2022. A similar persistence has recently been reported in Norway [10] and we expect that additional reports from other locations in Europe will likely draw the same picture. However, while the data from Norway indicate an important further increase in pediatric iGAS cases in 2023–2024, we find the opposite, with a moderate decrease in pediatric GAS hospitalizations in general and, specifically, in iGAS cases by approximately one-third compared with 2022–2023. This evolution mainly reflects a decrease in skin and soft tissue infections, bone, joint and muscle infections and pleural empyema, while suppurative GAS infections in the head and neck region (peritonsillar abscess, mastoiditis, orbital cellulitis, etc.) with or without bacteremia and cases with septic/toxic shock remained essentially stable. Our data are unsuitable to identify potential reasons for this divergent development, but the overall decrease in both iGAS and bacteremia cases may herald that the outbreak is on the decline. Hypothetical explanation may include a recovering population immunity against GAS in general or against specific outbreak clones. This would be supported by our observation that systemic GAS infections appear to decline more quickly than complications adjacent to the pharyngeal carriage site of GAS. Moreover, the observed decline of severe respiratory disease, i.e., mostly pleural empyema, could be explained by the epidemiology of influenza. Under the assumption that a preceding influenza virus infection is a major risk factor for complicated lower respiratory tract GAS disease [6,11], the poor overlap of the 2023–2024 GAS and influenza epidemic curves, respectively, may have restricted the risk phase for pleural empyema to a shorter period than in 2022–2023 (Figure 1C). For instance, Lassoued and co-workers similarly observed a coincidence of GAS pleural empyema and influenza activity in 2022–2023 in France and were able to demonstrate that the majority of pleural empyema patients were indeed co-infected with influenza virus [12]. A rather specific role of influenza restricted to the pathogenesis of GAS pleural empyema—but not other GAS disease—could also explain observations indicating that non-invasive GAS infections in the 2022–2023 outbreak year did not appear to be related to the circulation of influenza, e.g., in Portugal [13].

Our study has several limitations. Apart from the single-site design, which reflects the epidemiology of a demographically stable but geographically small area, the case figures in the subgroup analyses were small, allowing for wide margins of error. The data on viral co-circulation refer to the shapes of the respective epidemic curves, but not the co-occurrence of pathogens in the individual patients at the time of GAS infection.

In conclusion, however, our observations spanning 11 years of GAS hospitalizations in patients below 16 years of age demonstrate that the outbreak of severe GAS disease beginning in late 2022 continued through June 2024, albeit with a decline in cases in the second winter season. The decline mainly affected distant body sites and systemic infections, while the classic suppurative complications in the head area remained largely unchanged.

## Figures and Tables

**Table 1:** Clinical characteristics of patients below 16 years of age admitted because of a group A streptococcal infection to the in-patient service of the Departments of Pediatrics or Pediatric Surgery, Bern University Hospital, Switzerland, between 1 July 2013 and 30 June 2024.

		Observation Period			
Variable	2013–2024	2013–2020	2022–2023	2023–2024	*p* Value
*Demographics*					
No. of cases (n)	332	161	89	63	
Female sex (n, %)	130 (39)	65 (40)	32 (36)	25 (40)	
Median age, years [IQR]	5.4 [2.9–8.6]	5.6 [3.3–9.7]	4.9 [2.4–8.2]	4.7 [2.9–8.9]	0.101 ^1^
*Clinical manifestations*					
Median delay from onset to admission, days [IQR]	4.0 [2.0–7.0]	3.0 [2.0–6.0]	4.0 [3.0–7.0]	4.0 [2.8–6.0]	0.015 ^1^
Primary organ involvement of GAS infection (n, %)					
Head	203 (61)	99 (61)	48 (54)	45 (71)	
Skin and soft tissue	67 (20)	40 (25)	15 (17)	5 (8)	
Lower respiratory tract	30 (9)	10 (6)	13 (15)	7 (11)	
Bone, joint, muscle	18 (5	5 (3)	10 (11)	2 (3)	
Systemic onset	12 (4)	5 (3)	3 (3)	4 (6) ^2^	
Central nervous system	2 (0.6)	2 (1.2)	0	0	
*Severity (n, %)*					
GAS bacteremia	42 (13)	16 (10)	15 (17)	11 (17)	0.004 ^1^
Invasive GAS disease (iGAS)	91 (27)	35 (22)	32 (36)	21 (33)	0.010 ^1^
Septic/toxic shock	35 (11)	16 (10)	8 (9)	10 (16)	0.590 ^1^
*Outcome*					
Median length of stay, days [IQR]	5.0 [3.0–7.0]	5.0 [3.0–7.0]	5.0 [3.0–7.0]	4.0 [3.0–7.0]	0.368 ^2^
Intensive Care Unit (ICU) admission (n, %)	50 (15)	25 (16)	13 (15)	13 (21)	0.568 ^3^
Use of inotropes (n, % of ICU patients)	25 (50)	11 (40)	6 (46)	9 (69)	0.383 ^3^
Mechanical ventilation (n, % of ICU patients)	25 (8)	13 (8)	6 (46)	6 (46)	0.919 ^3^
Surgical intervention(s) (n, %)	241 (74)	117 (73)	67 (75)	43 (68)	0.708 ^3^
Death (n, %)	1 (0.3)	1 (0.6)	0	0	

^1^ In 2013–2020 vs. 2022–2024; ^2^ includes 1 case of primary GAS peritonitis; ^3^ 2013–2020 vs. 2022–2023 vs. 2023–2024.

## Data Availability

The data presented in this study are available upon well-founded request from the corresponding author.

## Supplementary Materials

The following supporting information can be downloaded at: https://www.mdpi.com/article/10.3390/idr16050067/s1, Table S1. Definition of invasive group A streptococcal disease (iGAS); Figure S1. Monthly hospitalization figures for all GAS infections (grey bars), GAS lower30 respiratory tract disease (green bars), and detection rates of Respiratory Syncytial Virus31 (blue line) and SARS-CoV-2 (red line) at the Departments of Pediatrics and Pediatric32 Surgery, Bern University Hospital, Switzerland, between 1 July 2022 and 30 June 2024.
