# Supplementary material for: Ongoing Excess Hospitalizations for Severe Pediatric Group A Streptococcal Disease in 2023–2024—A Single-Center Report
Source: Infect Dis Rep. 2024 Sep 2;16(5):864–9. doi: 10.3390/idr16050067 (PMC11417816; doi:10.3390/idr16050067)
Supplement: Supplementary file 1 [file idr-16-00067-s001.zip › idr-3131841-supplementary.pdf]

Supplementary data file

Ongoing excess hospitalizations for severe pediatric group A streptococcal disease in 2023-2024 – a single center report

Nina Schöbi, Andrea Duppenthaler, Matthias Horn, Andreas Bartenstein, Kristina Keitel,[3] Matthias V Kopp, Philipp KA Agyeman, Christoph Aebi

Table S1. Definition of invasive group A streptococcal disease (iGAS)

Identification of group A streptococci (*Streptococcus pyogenes)* in a normally sterile body site (culture, PCR, antigen test*) such as blood, cerebrospinal fluid, aspirate from a body cavity (e.g., pleural or pericardial space, joint), or surgically sampled deep tissue (e.g., muscle or bone)

OR

Severe clinical presentation** without alternative diagnosis AND identification of GAS from a non-sterile site (culture, PCR, antigen test).

*may also include *Streptococcus dysgalactiae* subsp. *equisimilis*

**Severe clinical presentation consists of one of the following:

(1) Toxic shock syndrome [1] with arterial hypotension (systolic blood pressure < 5th Percentile for age, see Table below) PLUS ≥ 2 of the following criteria

  a. Renal impairment (creatinine > 2x the upper limit of normal range for age); b) coagulopathy (platelet count < 100 G/L or clinical signs of disseminated intravascular coagulation (DIC).
  b. Liver impairment (ALAT, ASAT or bilirubin > 2 x the upper limit of normal for age).
  c. Generalized erythema with or without subsequent desquamation.
  d. ARDS (acute respiratory distress syndrome).

(2) Necrotizing fasciitis

Figure S1. Monthly hospitalization figures for all GAS infections (grey bars), GAS lower respiratory tract disease (green bars), and detection rates of Respiratory Syncytial Virus (blue line) and SARS-CoV-2 (red line) at the Departments of Pediatrics and Pediatric Surgery, Bern University Hospital, Switzerland, between 1 July 2022 and 30 June 2024.

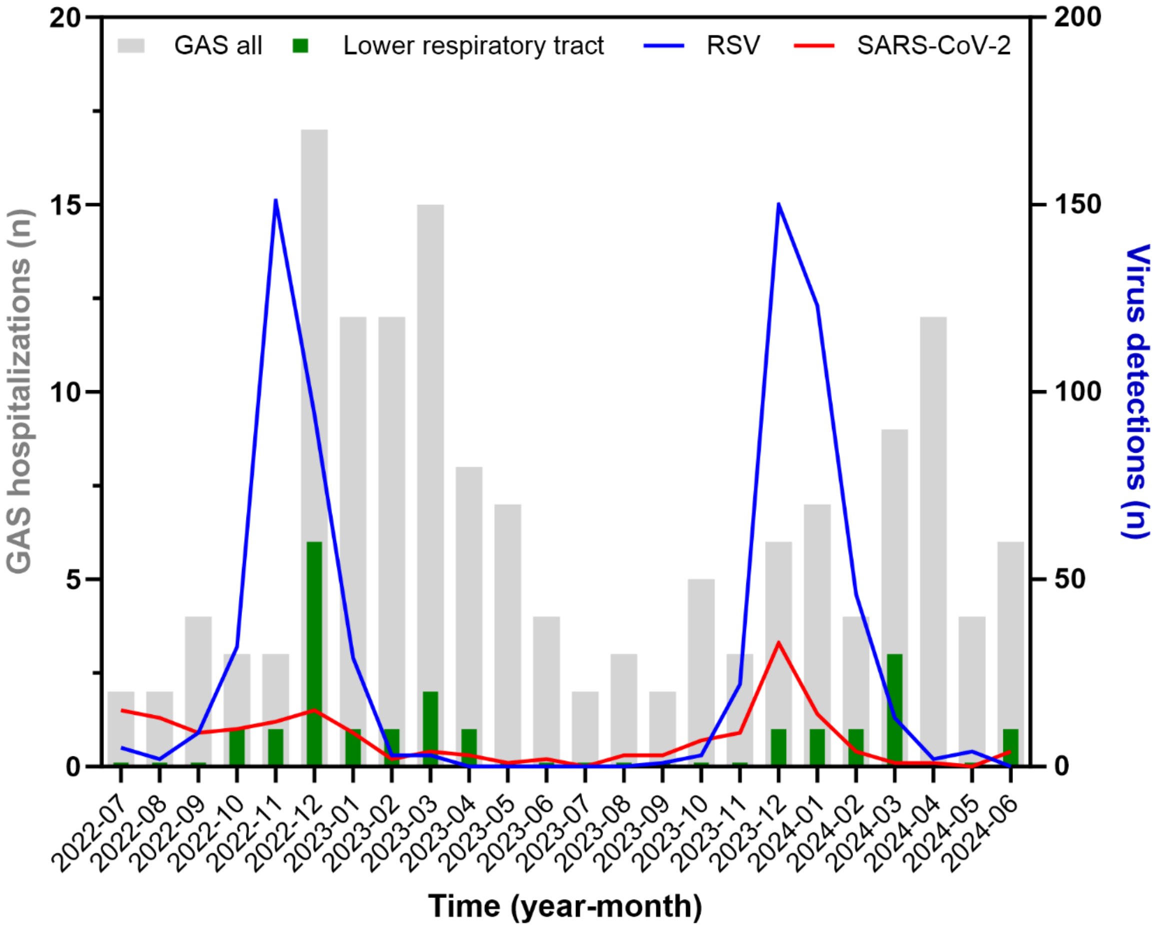

References

1. Defining the group A streptococcal toxic shock syndrome. Rationale and consensus definition. The Working Group on Severe Streptococcal Infections. *JAMA* **1993**, *269*, 390-391.
